# Vitamin D deficiency in pregnancy and its impact on the fetus, the newborn and in childhood

**DOI:** 10.1016/j.rpped.2014.05.004

**Published:** 2015-03

**Authors:** Marilyn Urrutia-Pereira, Dirceu Solé

**Affiliations:** a Pontificia Universidade Católica do Rio Grande do Sul, Porto Alegre, RS, Brazil; b Universidade Federal de São Paulo, São Paulo, SP, Brazil

**Keywords:** Vitamin D, Pregnancy, Lactation, Fetus, Newborn, Children, Fetal programming

## Abstract

**OBJECTIVE:**

Vitamin D deficiency (VDD) in pregnant women and their children is an important health problem with severe consequences for the health of both. Thus, the objectives of this review were to reassess the magnitude and consequences of VDD during pregnancy, lactation and infancy, associated risk factors, prevention methods, and to explore epigenetic mechanisms in early fetal life capable of explaining many of the non-skeletal benefits of vitamin D (ViD).

**DATA SOURCE:**

Original and review articles, and consensus documents with elevated level of evidence for VDD-related clinical decisions on the health of pregnant women and their children, as well as articles on the influence of ViD on epigenetic mechanisms of fetal programming of chronic diseases in adulthood were selected among articles published on PubMed over the last 20 years, using the search term *VitD status*, in combination with *Pregnancy*, *Offspring health*, *Child outcomes*, and *Programming*.

**DATA SYNTHESIS:**

The following items were analyzed: ViD physiology and metabolism, risk factors for VDD and implications in pregnancy, lactation and infancy, concentration cutoff to define VDD, the variability of methods for VDD detection, recommendations on ViD replacement in pregnant women, the newborn and the child, and the epigenetic influence of ViD.

**CONCLUSIONS:**

VDD is a common condition among high-risk pregnant women and their children. The routine monitoring of serum 25(OH)D3 levels in antenatal period is mandatory. Early preventive measures should be taken at the slightest suspicion of VDD in pregnant women, to reduce morbidity during pregnancy and lactation, as well as its subsequent impact on the fetus, the newborn and the child.

## Introduction

Vitamin D deficiency (VDD) is identified as a public health problem in many countries, and pregnant women have been identified as a high-risk group, among whom the prevalence of VDD ranges between 20 and 40%.^1^

While it is acknowledged that vitamin D (ViD) supplementation is effective in preventing the VDD, many children are born with this deficiency, raising questions as to how and why VDD affects the pregnancy, the fetus and the newborn's health.^2^

The increase in the number of studies on this subject shows conflicting results on the association between 25(OH)D levels in pregnancy and adverse effects on maternal and fetal health, both skeletal and non-skeletal (autoimmune diseases, cardiovascular diseases, diabetes and certain types of cancer through "fetal imprinting").^3^ Thus, it is advisable to review VDD in mothers and their children so that strategies can be implemented to prevent VDD in pregnancy and lactation, in order to prevent its impact on the fetus, the newborn and in childhood, aiming at a possible reduction in the future development of chronic diseases in adulthood.

## Method

PubMed database was used for the selection of the articles used in this review, and the evaluated search period comprised the last 20 years. The following search terms were used: *VitD status* alone and in combination with the words: Pregnancy, Offspring health, Child outcomes, Programming. Among the identified studies, case reports and intervention studies without randomization were excluded. Original articles, review articles and consensuses with high level of evidence for clinical decisions related to VDD regarding the health of pregnant women and their children were selected. Moreover, we selected articles that evaluated the influence of ViD in the epigenetic mechanisms of fetal programming of chronic diseases in adulthood, focusing on the latest works. The most relevant articles according to the objectives of this review were therefore chosen.

## Physiology and vitamin D metabolism

**Figure 1:**
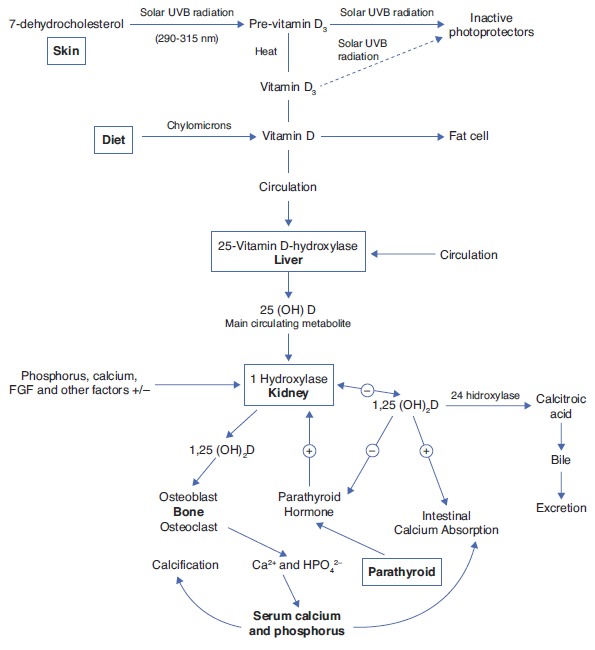
Synthesis and metabolism of vitamin D as well as its action on the regulation of levels of calcium, phosphorus and bone metabolism (*Adapted* from *Holick MF*.^8^). UVB, ultraviolet light B; 25 (OH) D, 25-hydroxyvitamin D; 1,25 (OH) 2D, 1,25-dihydroxyvitamin D; 23-FGF, fibroblast growth factor 23; Ca2 +, calcium ions; HPO42-, phosphorus ions

There are two sources of ViD for humans. An exogenous one is provided by the diet in the form of vitamins D2 and D3. In the endogenous production, cholecalciferol (D3), the main source of ViD, is synthesized in the skin by the action of ultraviolet B (UVB) radiation through the photolysis of 7-dehydrocholesterol and transformed into vitamin D3. Sufficient exposure to sunlight or UVB radiation is up to 18IU/cm^2^ in 3 hours. This process takes place in two phases: the first one occurs in the deep layers of the dermis and consists in the photo conversion of 7-dehydrocholesterol into pre-vitamin D or pre-calciferol (Fig. 1).^4^

In the second phase, there is a chemical isomerization depending on body temperature, and pre-vitamin D slowly and progressively turns into vitamin D3, which has high affinity for the ViD carrier protein (DBP), and the pre-vitamin D, with lower binding affinity, remains in the skin.^4^ Upon reaching the skin capillary network, ViD is transported to the liver and binds with DBP, where it starts its metabolic transformation.^4^

The two types of ViD undergo complex processing to be metabolically active.^5^ Initially, the pre-hormone is hydroxylated in the liver at the carbon 25 position through the action of vitamin D-25-hydroxylase 1a (1-OHase), which constitutes an enzyme system dependent on cytochrome P-450 (CYP27B) present in liver microsomes and mitochondria, and originates 25-hydroxyvitamin D (25(OH)D), the most abundant circulating form of ViD.^4^ Its mean blood concentration is 20-50ng/mL (50-125nmol/L) and it has an average life of approximately 3-4 weeks.^4^ It is estimated that its circulating pool is in dynamic equilibrium with reserves of 25(OH)D (muscle and adipose tissue), which makes blood levels a reliable indicator of the state of the ViD reserves in the body.^4^ Under normal circumstances, the percentage of conversion into 25(OH)D is low, with a distribution of almost 50% in the fat and muscle compartments. When there is excess intake of ViD, most of it is stored in the fatty deposits.^4^

As 25(OH)D has low biological activity, it is transported to the kidney where it undergoes the second hydroxylation, and then the active forms are obtained: calcitriol (1a-dihydroxyvitamin D) (1.25(OH)_2_D) and 24.25-dihydroxyvitamin D (24.25(OH)_2_D), through the respective action of enzymes 1-OHase and vitamin D-24-hydroxylase (24-OHase) present in mitochondria of cells of the proximal convoluted tubule.^5^

DBP and 25(OH)D are filtered by the glomerulus and absorbed in the proximal tubule by low-density lipoprotein receptors, which regulate the uptake of the 25(OH)D-DBP complex within the tubule cells and the subsequent hydroxylation to 1.25(OH)_2_D.^4^

1-OHase is also found in other tissues that express ViD receptors, such as the placenta, colon, activated mononuclear cells and osteoblasts, which could produce 1.25(OH)_2_D with local autocrine or paracrine function.^6^

Several factors regulate the levels of 1.25(OH)_2_D: 1-OHase, whose hydroxylation is activated by the parathormone (PTH), and calcitonin, which is inhibited by serum levels of calcium, phosphorus and 1.25(OH)_2_D itself, and whose average life is 15 days.^6^

Blood levels of phosphorus have a direct action, without the intervention of PTH, and hypophosphatemia increases the production of 1.25(OH)_2_D.

**Figure 2:**
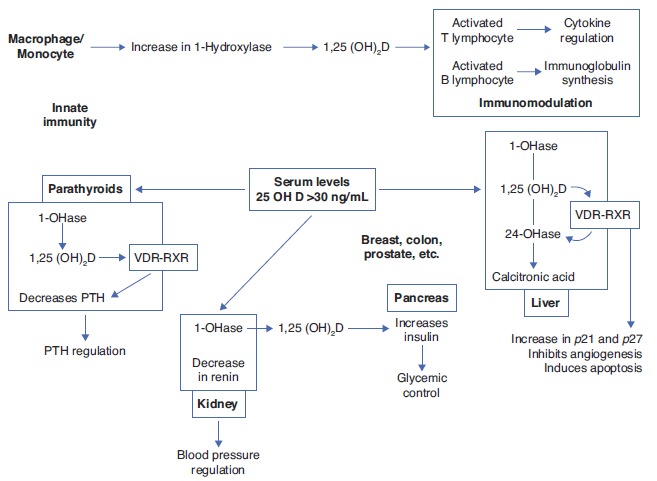
Non-skeletal functions of 1.25-dihydroxyvitamin D (*Adapted from Holick MF*.^8^). VDR, vitamin D receptor; RXR, target region; 1-OHase, 25-hydroxyvitamin D-1a-hydroxylase; 25 (OH) D: 25-hydroxyvitamin D; 1,25 (OH) 2D, 1,25-dihydroxyvitamin D; 24-OHase, 25-hydroxyvitamin D-24-hydroxylase; p21 and p27, genes involved in the control of proliferation, angiogenesis inhibition and cell apoptosis

**Figure 3:**
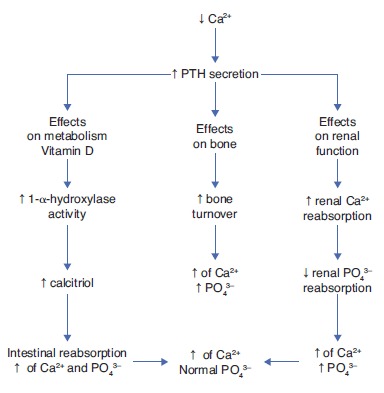
Regulatory mechanisms of serum levels of calcium and phosphorus. *Adapted from Ross AC et al*.^7^ PTH, parathyroid hormone; Ca^2+^, calcium ion; PO_4_^3-^, phosphate ions; ↑, increase; ↓, decrease

Thus, in addition to the main action of ViD in maintaining physiological levels of calcium and phosphorus capable of allowing metabolism, neuromuscular transmission and bone mineralization, the presence of ViD receptors in bone, bone marrow, cartilage, hair follicle, adipose tissue, adrenal gland, brain, stomach, small intestine, distal kidney tubule, colon, pancreas (B cells), liver, lung, muscle, activated B and T lymphocytes, heart cells, vascular smooth muscle cells, gonads, prostate, retina, thymus and thyroid glands has been described, which reinforce such diverse and important ViD functions (Fig. 2).^5^ Figure 3 summarizes the mechanisms involved in the control of serum calcium and phosphorus levels.^7^

## Risk factors for ViD deficiency

The main source of ViD for children and adults is exposure to sunlight, so the main cause of VDD is the decrease of its endogenous production. Any factor that affects the transmission of UVB radiation or interferes with its skin penetration will determine the reduction of 25(OH)D.^4^

Among these risk factors are:

- Use of sunscreen with a protection factor of 30 reduces the synthesis of ViD in the skin, above 95%
- Individuals with darker skin have natural sun protection, as melanin absorbs UVB radiation, and thus they need 3-5 times longer sun exposure to synthesize the same amount of ViD than individuals with light skin
- Skin aging as well as age decrease the capacity of the skin to produce ViD due to lower availability of 7-dehydrocholesterol
- Skin damage such as burns decrease ViD production
- Atmospheric contamination and overcast may act as sunscreen
- The season of the year and the time of the day influence dramatically on the skin production of ViD

The second cause is the reduced intake of ViD, as few foods contain high quantities of it (blue fish, egg yolks). The intake of the vitamin can be increased with fortified products such as dairy products, although the amount of ViD they provide may be insufficient for an adequate state of ViD.^8^

Obesity can also be associated to VDD, because being a fat-soluble vitamin, ViD is sequestered by body fat. Another factor is the malabsorption of fats, as it occurs with the use of bile acid chelating agents (cholestyramine), in cystic fibrosis, celiac disease and Crohn's disease, among others.^8^ Also, anticonvulsants, glucocorticoids and drugs used in HIV treatment can lead to VDD by increasing the hepatic expression of cytochrome P-450 and the catabolism of 25(OH)D. In severe liver failure, chronic granulomatous disease, certain lymphomas and primary hypoparathyroidism, patients have increased metabolism of 25(OH)D into 1.25 (OH)_2_D, and thus a high risk of VDD.^8^

## Vitamin D deficiency in pregnancy and fetal programming

During fetal life, the body tissues and organs go through critical development periods that coincide with periods of rapid cell division.^9^ Fetal programming is a process through which a stimulus or insult, during a certain development period, would have effects throughout life.^10^ This term is used to describe the mechanisms that determine fetal adaptation to changes that accompany the gene-environment interaction during specific periods of fetal development.^9^

It has been demonstrated that nutritional and environmental exposures during these sensitive periods of life may influence fetal growth and the development of physiological functions of organs and systems. Permanent changes in many physiological processes of this programming can modify the expression patterns of genes, with consequent influence on phenotypes and functions (epigenetic mechanisms).^11^

Thus, the closer to fertilization these changes take place, the greater the potential for epigenetic changes and their correspondence in newborns to occur in response to environmental changes. These changes in placenta/embryo/fetus provide a plausible explanation for the concept of fetal origin of adult diseases.^12^

It is currently recognized that nutrition in early life and other environmental factors play a key role in the pathogenesis and predisposition to diseases, which seem to propagate to subsequent generations. Epigenetic modifications establish a link with the nutritional status during critical periods of development and cause changes in gene expression that can lead to the development of disease phenotypes.^13^

Recent evidence indicates that nutrients can modify the immune and metabolic programming during sensitive periods of fetal and postnatal development. Thus, modern diet patterns could increase the risk of immune and metabolic dysregulation associated with the increase of a wide range of noncommunicable diseases.^11^ Among these nutrients, ViD is emphasized, and its effects on fetal programming and gene regulation might explain why it has been associated with many health benefits throughout life.^8^^,^ 14 ^,^ 15

There seems to be a window of early development in life that can shape the nature of the immune response in adulthood, and thus early life factors that predispose individuals to chronic lung disease would not be limited to the post-natal period, as evidence indicates that there are intrauterine effects such as maternal smoking, diet and ViD that influence the development of the lung and the subsequent development of asthma and chronic obstructive pulmonary disease.^16^^,^ 17

As much of the reprogramming that occurs during childhood may go unnoticed until adulthood, the better understanding of the interaction between genetics and epigenetics in critical time windows of development would improve our capacity to determine individual susceptibility to a wide range of diseases.^13^ Although these epigenetic changes appear to be potentially reversible, little is known about the rate and extent of improvements in response to positive environmental changes, including nutrition, and to what extent they depend on the duration of exposure to a deficient maternal environment also remains unknown.^18^

Thus, it can be observed that, in spite of all this new range of information, maternal nutrition has received little attention in the context of implementation of effective prevention goals (MDG, Millennium Development Goals). This could be attributed to the lack of a solid and strong foundation to justify the enormous effort required to improve the nutritional status of all women of reproductive age.^19^ To elucidate the true role of nutritional epigenetics^13^^,^ 14 in fetal programming of pregnant women, especially those with VDD, would allow the use of effective prevention measures to improve maternal and fetal health and prevent the development of future chronic diseases.

## Vitamin D and calcium metabolism in pregnancy

During pregnancy and lactation, significant changes in calcium and ViD metabolism occur to provide for the needs required for fetal bone mineralization. In the first trimester, the fetus accumulates 2-3mg/day of calcium in the skeleton, which doubles in the last trimester.^1^

The pregnant woman's body adapts to the fetal needs and increases calcium absorption in early pregnancy, reaching a peak in the last trimester.^1^ The transfer is counterbalanced by increased intestinal absorption and decreased urinary excretion of calcium.

Plasma levels of 1.25(OH)_2_D increase in early pregnancy, reaching a peak in the third trimester and returning to normal during lactation. The stimulus for increased synthesis of 1.25(OH)_2_D is unclear, considering that PTH levels do not change during pregnancy.^1^

A potent stimulus to placental transfer of calcium and placental synthesis of ViD is the PTH-related peptide (PTHrP), produced in the fetal parathyroid and placental tissues, which increases the synthesis of ViD.^1^ The PTHrP can reach the maternal circulation and it acts through the PTH/PTHrP receptor in the kidney and bones, being a mediator in the increase of 1.25(OH)_2_D and helping in the regulation of calcium and PTH levels in pregnancy.^1^

Other signals involved in the regulation process include prolactin and the placental lactogen hormone, which increase intestinal calcium absorption, reduce urinary calcium excretion and stimulate the production of PTHrP and 1.25(OH)_2_D. Moreover, the increase in the maternal blood levels of calcitonin and osteoprotegerin protects the mother's skeleton from excessive calcium resorption.^1^

Additionally, during lactation, there is a relative estrogen deficiency, caused by elevated levels of prolactin, which determines bone resorption and suppression of PTH levels. PTHrP levels are elevated and act as a substitute for PTH, while maintaining urinary calcium absorption and bone resorption.^1^

## Implications of vitamin D deficiency in pregnancy

Recent studies emphasize the importance of non-classical roles of ViD during pregnancy and in the placenta and correlate VDD in pregnancy with preeclampsia, insulin resistance, gestational diabetes, bacterial vaginosis and increased frequency of cesarean delivery.^20^

ViD supplementation reduces the risk of preeclampsia. Studies in women with preeclampsia have shown low urinary excretion of calcium, low ionized calcium levels, high levels of PTH and low levels of 1.25(OH)_2_D.^21^ An association between maternal VDD (<50nmol/L) and increased risk of gestational diabetes (OR:2.66, 95% CI: 1.01 to 7.02),^22^ as well as the fact that VDD is an independent risk factor for bacterial vaginosis in pregnancy^23^ have also been documented. A recent randomized and controlled study showed that supplementation with 4,000IU/d during pregnancy was associated with reduced risk of combined morbidities, such as maternal infections, cesarean section and preterm delivery.^21^^-^ 24

A prospective study showed that cesarean delivery is four times more common in women with VDD (<37.5nmol/L) when compared to women with normal levels of ViD (OR: 3.84, 95% CI: 1.71 to 8.62).^25^

## Implications of vitamin D deficiency in lactation and childhood

Adequate levels of ViD are also important for the health of the fetus and the newborn, and poor skeletal mineralization *in utero* due to VDD can be manifested in the newborn as congenital rickets, osteopenia or craniotabes.^1^

Maternal VDD is one of the main risk factors for VDD in childhood, as in the first 6-8 weeks of life newborns depend on the ViD transferred across the placenta while in the womb. This association is linear,^26^ and the 25(OH)D levels of the newborn correspond to 60-89% of maternal values.^2^

These levels decrease on the 8^th^ week and, therefore, exclusively breastfed infants have an increased risk of VDD, as human milk has a low concentration of ViD (approximately 20-60IU/L; 1.5-3% of the maternal level). This concentration is not sufficient to maintain optimal levels of ViD, especially when exposure to sunlight is limited,^27^ and may induce seizures caused by hypocalcemia and dilated cardiomyopathy.^1^

Observational studies have shown that low levels of ViD during pregnancy and VDD in childhood are related to the increase in other non-skeletal manifestations,^2^ such as a higher incidence of acute lower respiratory tract infections and recurrent wheezing in the first five years of life.^28^

Contradictory results are observed in relation to the increased risk of allergic diseases such as asthma, eczema and rhinitis in the presence of VDD.^29^ However, a cohort study showed increased asthma and eczema among children whose mothers had high serum levels of 25(OH)D during pregnancy.^30^

Japanese schoolchildren that received ViD supplementation (1,200IU/d) had a 42% reduction in the incidence of type A Influenza.^1^ A cohort study showed that supplementation with 2,000IU/d of ViD during the first year of life was associated with a reduction in the incidence of type I diabetes during a 30-year follow up.^31^

These results show the importance of maintaining adequate levels of ViD in fetal life and in early life and childhood.

## Vitamin D deficiency, insufficiency and sufficiency

The cutoff point to define the ViD status based on the values of 25(OH)D is debatable. There are currently two criteria:

- The Committee of the Institute of Medicine (IOM, USA)^32^ considers values lower than 20ng/mL (50nmol/L) as indicators of VDD, with 10ng/mL (25nmol/L) being considered severe VDD, and 10-19ng/mL (25-49nmol/L) being considered ViD insufficiency. ViD levels <10ng/mL are associated with rickets and osteomalacia in adults and children. Between 10-19ng/mL, there is increased rate of bone resorption and increased risk of secondary hypoparathyroidism. Thus, the IOM recommends a threshold level of 20ng/mL as adequate to maintain bone health at all ages
- The Endocrine Society (USA) proposes VDD in the presence of ViD levels inferior to 20ng/mL and ViD insufficiency between 20-30ng/mL (50-75nmol/L).33 In clinical practice, a patient would have sufficient levels when the concentration of 25(OH)D were greater than 30ng/mL. Several authors support this concentration cutoff for musculoskeletal health and mineral metabolism (prevention of rickets and osteomalacia, elevated PTH levels, osteoporotic fractures and falls among the elderly)^34^

The main differences between the IOM^32^ and the Endocrine Society^33^ are the overall health endpoints. The IOM makes recommendations to ensure skeletal health and suggests there is lack of evidence to support recommendations of potential non-skeletal benefits of ViD, considering that individuals with levels inferior to 20ng/mL are not deficient, as 97% of individuals with these levels have adequate bone health.^32^

The Endocrine Society^33^ considers that serum levels superior to 30ng/mL bring greater benefits to health in general, when compared to a level of 20ng/mL, and that skeletal health is not guaranteed with levels inferior to 30ng/mL; these data are supported by three supposed observations:

- The increase in PTH reaches a plateau when serum 25(OH)D is ≥30ng/mL;
- There is a decrease in the risk of fractures in individuals with levels ≥30ng/ml;
- Calcium absorption is maximal for serum levels of 30ng/mL.

## Detection method

Serum levels of 25(OH)D are the best indicators of ViD status; however, methodological issues limit comparisons between studies, as well as the adoption of cutoffs to define hypovitaminosis D.^4^

Considering the method employed, it is important to ask: 1) Does the method quantify the actual level of ViD? and 2) Are these results reproducible and comparable between laboratories?^4^

Liquid Chromatography Coupled with Mass Spectrometry (LC-MS/MS) was recommended as the preferred method by the National Diet and Nutrition Survey.^34^ In general, all available methods are valid to detect severe VDD. As for moderate deficiency, there is a risk of error, which can be reduced by considering the reference values of each laboratory; however, for research studies, the methods employed should be standardized.^6^

## Recommendations

If the main source of ViD comes from sunlight exposure, it is difficult to establish generalized requirements for the intake, especially due to the many variables associated with its deficiency.^6^

Table 1 shows the different recommended daily doses of ViD. Although the IOM recommends a daily intake of 200IU of ViD, this was insufficient to keep concentrations of 25(OH)D above 50nmol/L.^35^ On the other hand, while recognizing that the exclusion of habitual sunlight exposure is a risk for VDD, it is unknown what level of exposure is safe and sufficient to maintain adequate levels of ViD.^35^ Table 2 shows the different contents of ViD-fortified and non-ViD-fortified foods available for consumption in the United States. In Brazil, such data are scarce and most often do not reflect the content of all available processed foods.

**Table 1:** Dietary reference intakes and maximum tolerable intakes of vitamin D in different stages of life - IOM, 2010.^32^

Age	Vitamin D mcg/day			
	AI	EMR	RDI	MTI
Infants (0 to 6 months)	10 (400 UI)	—	—	25 (1,000 UI)
6 to 12 months	10 (400 UI)		15 (600 UI)	38 (1,520 UI)
13 months to 3 years	—	10 (400 UI)	15 (600 UI)	63 (2,520 UI)
4 to 8 years	—	10 (400 UI)	15 (600 UI)	75 (3,000 UI)
Men: 9 to 70 year	—	10 (400 UI)	20 (800 UI)	100 (4,000 UI)
Women: 9 to 70 years	—	10 (400 UI)	15 (600 UI)	100 (4,000 UI)
Age >70 years	—	10 (400UI)	20 (800 UI)	100 (4,000 UI)
Pregnancy (14 to 50 years)	—	10 (400 UI)	15 (600 UI)	100 (4,000 UI)
Lactation (14 to 50 years)	—	10 (400 UI)	15 (600 UI)	100 (4,000 UI)

AI, intake adequate; EMR, estimated mean requirement; RDI, Recommended daily intake; MIT, maximum tolerable intake level; between brackets, the corresponding value in international units (IU).

**Table 2:** Dietary sources of vitamins D_2_ and D_3_. ^7^

Source	Vitamin D content
Salmon	
Wild – 100g	600-1000UI de Vit D_3_
Bred in captivity – 100g	100-250UI Vit D_3_ ou D_2_
Canned – 100g	300-600UI Vit D_3_
Canned sardines – 100g	~300 UI Vit D_3_
Canned Horsetail – 100g	~250 UI Vit D_3_
Canned tuna – 100g	~230 UI Vit D_3_
Cod-liver oil (1 tbsp)	~400-1000 UI Vit D_3_
Fresh Shiitake mushroom – 100g	~100 UI Vit D_3_
Dried Shiitake mushroom – 100g	~1600 UI Vit D_3_
Egg yolk	~20 UI Vit D_3_ ou D_2_
Fortified foods	
Fortified milk – 240mL	~100 UI Vit D_3_
Orange juice – 240mL	~100 UI Vit D_3_
Infant formulas – 240mL	~100 UI Vit D_3_
Fortified yogurts – 240mL	~100 UI Vit D_3_
Fortified butter – 100g	~50 UI Vit D_3_
Fortified margarine – 100g	~430 UI Vit D_3_
Fortified cheese – 85g	~100 UI Vit D_3_
Fortified morning cereals – meal	~100 UI Vit D_3_

In most countries, the monitoring of serum levels of 25(OH)D during pregnancy is not performed; however, it is recommended that women with one or more risk factors for VDD be monitored in early and mid-pregnancy.^36^ Consequently, the risk of VDD during pregnancy would be reduced, as well as the negative effects on the mother and the fetus; however, the appropriate dose of ViD supplementation for pregnant women to prevent VDD remains unknown.

Few studies have evaluated ViD supplementation in pregnancy, as well as the optimal levels to be offered. Several factors hinder the observation of an adequate dose-response between low 25(OH)D levels and clinical outcomes: lack of data with extreme serum 25(OH)D levels and the wide variety of studied subjects (diversity of location, latitude, season, ethnicity, body mass index, type of diet, lifestyle, skin pigmentation, family history of metabolic complications in pregnancy, physical activity and method used to quantify 25(OH)D).^1^

A meta-analysis of studies carried out in adults on ViD supplementation (2,000IU/d) and bone health showed that for each 1IU of vitamin D3 ingested, there is a corresponding increase of 0.016nmol/L in serum levels of 25(OH)D.^37^ Despite the limited evidence on the effects of ViD supplementation in pregnancy and the outcomes in the mother's health and perinatal and early childhood effects, ViD supplementation (800-1,000IU/d) was accompanied by a protective effect in newborns with low birth weight.^9^^,^ 38

The Canadian Academy of Pediatrics (CAP)^37^ recommends supplementation with 2.000IU/d during pregnancy and lactation.^38^ According to the American College of Obstetricians and Gynecologists,^38^ in the presence of VDD diagnosed during pregnancy, there should be supplementation with 1.000-2.000IU/day of ViD.

Studies have shown that maternal exposure during pregnancy to serum levels of 25(OH)D superior to 75nmol/L had no effect on the intelligence and psychological health of the children or on their cardiovascular system, but it could increase the risk of atopic diseases.^30^

In summary, ViD serum levels in pregnancy are a major concern, and the prevention of VDD in pregnant women and their newborns is vital and urgent.

## Vitamin D recommendations for the newborn and children

The Canadian Academy of Pediatrics defines ViD needs during the first year of life as 200IU/d for preterm newborns and 400IU/d for other children. However, would the weight gain observed in the first year of life be accompanied by increased needs of ViD in a weight-dependent mode?^38^ Moreover, the CAP also recommends that infants and children be exposed to sunlight for short periods - probably less than 15 minutes.^38^

The American Academy of Pediatrics recommends that children who are exclusively breastfed should receive supplementation with 400IU/day of ViD soon after birth and continue to receive during their development up to adolescence.^5^ Concerned about the bone health of premature infants, they recommend biochemical monitoring of their 25(OH)D levels during hospitalization, and recommend 200-400IU/d of ViD, both during hospitalization and after discharge.^39^^,^ 40 Recently, the IOM recommended 400IU/d for children younger than one year and 600IU/d for children aged between 1-8 years.^32^

## Conclusion

VDD in pregnant women and their children is a major health problem, with potential adverse consequences for overall health. Prevention strategies should ensure the ViD sufficiency in women during pregnancy and lactation. Evidence-based interventions to improve maternal and fetal nutrition, such as for ViD, are accompanied by a decrease of the impact on the health of their children.^41^

The ambiguities between the definitions of ViD status, combined with a lack of consistency in recommendations related to incorporation of routine testing of 25(OH)D levels in the prenatal period, especially in women with risk factors for VDD, dose and gestational age for the start of ViD supplementation, universal cutoffs for normal ViD values, lack of education about the benefits of ViD and the need for adequate sunlight exposure represent important barriers to the advance of the implementation of ViD supplemental guides, in order to improve this important health problem in pregnant women and their children in the short term. Large-scale studies in different geographical locations are necessary to identify the true role of ViD on the health of pregnant women and the "fetal imprinting" of their children.

## References

[1] Mulligan ML, Felton SK, Riek AE, Bernal-Mizrachi C (2010). Implications of vitamin D deficiency in pregnancy and
lactation. Am J Obstet Gynecol.

[2] Dawodu A, Wagner CL (2012). Prevention of vitamin D deficiency in mothers and
infants worldwide - a paradigm shift. Paediatr Int Child Health.

[3] Souberbielle JC, Body JJ, Lappe JM, Plebani M, Shoenfeld Y, Wang TJ (2010). Vitamin D and musculoskeletal health, cardiovascular
disease, autoimmunity and cancer: recomendations for clinical
practice. Autoimmun Rev.

[4] Chicote CC, Lorencio FG, Comité de Comunicación de la Sociedad Española de Bioquímica Clínica y
Patología Molecular (2013). Vitamina D: una perspectiva actual.

[5] Wagner CL, Greer FR, American Academy of Pediatrics Section on BreastfeedingAmerican Academy of
Pediatrics Committee on Nutrition (2008). Prevention of rickets and vitamin D deficiency in
infants, children, and adolescentes. Pediatrics.

[6] Masvidal Aliberch RM, Ortigosa Gómez S, Baraza Mendoza MC, Garcia-Algar O (2012). Vitamin D: pathophysiology and clinical applicability in
paediatrcs. An Pediatr (Barc)..

[7] Ross AC, Taylor CL, Yaktine AL, Del Valle HB, Institute of Medicine Committee to Review Dietary Reference Intakes for
Vitamin D and Calcium (2011). Overview of vitamin D.

[8] Holick MF (2007). Vitamin D deficiency. N Engl J Med.

[9] Cunninghan S, Cameron IT (2003). Consequences of fetal growth restriction during
childhood and adult life. Curr Obstet Gynecol.

[10] Kim YJ (2009). In utero programming of chronic disease. J Womens Med.

[11] Amarasekera M, Prescott SL, Palmer SL (2013). Nutrition in early life, imune-programming and
allergies: the role of epigenetics. Asian Pac J Allergy Immunol.

[12] McMillen IC, MacLaughlin SM, Muhlhausler BS, Gentili S, Duffield JL, Morrison JL (2008). Developmental origins of adults health and disease: the
role of periconceptional and fetal nutrition. Basic Clin Pharmacol Toxicol.

[13] Jang H, Serra C (2014). Nutrition, Epigenetics, and Diseases. Clin Nutr Res.

[14] Hossein-Nezhad A, Holick MF (2012). Optimize dietary intake of Vitamin D: an epigenetic
perspective. Curr Opin Clin Nutr Metab Care.

[15] Hossein-Nezhad A, Holick MF (2013). Vitamin D for health: a global
perspective. Mayo Clin Proc.

[16] Hykema MN, Blacuire MJ (2009). Intrauterine effects of maternal smoking on
sensitization asthma and chronic obstructive pulmonary disease. Proc Am Thorac Soc.

[17] Sharma S, Chhabra D, Kho AT, Hayden LP, Tantisira KG, Weiss ST (2014). The genomic origins of asthma. Thorax.

[18] Hambidge KM, Krebs NF, Westcott JE, Garces A, Goudar SS, Kodkany BS (2014). Preconception maternal nutrition: a multi-site
randomized controlled trial. BMC Pregnancy Childbirth.

[19] (2012). Shrimpton R: Global policy and programme guidance on
maternal nutrition: what exists, the mechanisms for providing it, and how to
improve them?. Paediatr Perinat Epidemiol.

[20] Kaushal M, Magon (2013). Vitamin D in pregnancy: a metabolic
outlook. Indian J Endocrinol Metab.

[21] Taufield PA, Ales KL, Resnick LM, Druzin ML, Gerther JM, Laragh JH (1987). Hypocalciuria in preeclampsia. N Engl J Med.

[22] Zhang C, Qiu C, Hu FB, David RM, van Dam RM, Bralley A (2008). Maternal plasma 25-hydroxyvitamn D concentration and the
risk for gestacional diabetes mellitus. PLoS ONE.

[23] Bodnar LM, Krohn MA, Simhan HN (2009). Maternal vitamin D deficiency is associated with
bacterial vaginose in the first trimester of pregnancy. J Nutr.

[24] Wagner CL, McNeil R, Hamilton SA, Winkler J, Rodriguez Cook C, Warner G (2013). A randomized trial of vitamin D supplementation in 2
community health center networks in South Carolina. Am J Obstet Gynecol.

[25] Merewood A, Mehta SD, Chen TC, Bauchner H, Holick MF (2009). Association between vitamin D deficiency and primary
cesarean section. J Clin Endocrinol Metab.

[26] Hillman LS, Haddad JG (1974). Human perinatal vitamin D metabolism I: 25-
Hydroxyvitamin D in maternal and cord blood. J Pediatr.

[27] American Academy of Pediatrics (2011). Policy statement - ultraviolet radiation: a hazard to
children and adolescents. Pediatrics.

[28] Camargo CA, Ingham T, Wickens K, Thadhani R, Silvers KM, Epton MJ (2011). Cord-blood 25-hydroxyvitamin D levels and risk of
respiratory infection, wheezing, and asthma. Pediatrics.

[29] Devereux G, Litonjua AA, Turner SW, Craig LC, McNeill G, Martindale S (2007). Maternal vitamin D intake during pregnancy and early
childhood wheezing. Am J Clin Nutr.

[30] Gale CR, Robinson SM, Harvey NC, Javaid MK, Jiang B, Martyn CN (2008). Maternal vitamin D status during pregnancy and child
outcomes. Eur J Clin Nutr.

[31] Hyppönen E, Läärä E, Reunanen A, Järvelin MR, Virtanen SM (2001). Intake of vitamin D and risk of type 1 diabetes: a
birth-cohort study. Lancet.

[32] Ross AC, Manson JE, Abrams SA, Aloia JF, Brannon PM, Clinton SK (2011). The 2011 report on dietary reference intakes for calcium
and vitamin D from the institute of medicine: what clinicians need to
know. J Clin Endocrinol Metab.

[33] Holick MF, Binkley NC, Bischoff-Ferrari HA, Gordon CM, Hanley DA, Heaney RP (2011). Evaluation, treatment, and prevention of vitamin D
deficiency: an Endocrine Society clinical practice guideline. J Clin Endocrinol Metab.

[34] De la Hunty A, Wallace AM, Gibson S, Viljakainen H, Lamberg-Allardt C, Ashwell M (2010). UK Food Standards Agency Workshop Consensus Report: the
choice of method for measuring 25-hydroxyvitamin D to estimate vitamin D status
for the UK national diet and nutrition survey. Br J Nutr.

[35] Ross AC, Taylor CL, Yaktine AL, Del Valle HB, Institute of Medicine Committee to Review Dietary Reference Intakes for
Vitamin D and Calcium (2011). Dietary reference intakes for calcium and vitamin D.

[36] Ponsonby AL, Lucas RM, Lewis RM, Halliday J (2010). Vitamin D status during pregnancy and aspects of
offspring. Nutrients.

[37] Thorne-Lyman A, Fawzi WW (2012). Vitamin D during pregnancy and maternal, neonatal and
infant health outcomes: a systematic review and meta-analysis. Paediatr Perinat Epidemiol.

[38] Canadian Paediatric Society (2007). Vitamin D supplementation: recommendations for Canadian
mothers and infants. Paediatr Child Health.

[39] ACOG Committee on Obstetric Practice (2011). ACOG committee opinion No.495: Vitamin D: screening and
supplementation during pregnancy. Obstet Gynecol.

[40] Abrams SA, Committee on Nutrition (2013). Calcium and vitamin D requirements of enterally fed
preterm infants. Pediatrics.

[41] Bhutta ZA, Das JK, Rizvi A, Gaffey MF, Walker N, Horton S (2013). Evidence-based interventions for improvement of maternal
and child nutrition: what can be done and at what cost?. Lancet.

