# Supplementary material for: Vitamin D deficiency in pregnancy and its impact on the fetus, the newborn and in childhood
Source: Rev Paul Pediatr. 2015 Mar;33(1):104–13. doi: 10.1016/j.rpped.2014.05.004 (PMC4436962; doi:10.1016/j.rpped.2014.05.004)
Supplement: Translation

# Translation: Deficiência de vitamina D na gravidez e o seu impacto sobre o feto, o recém-nascido e na infância

**Authors:** Marilyn Urrutia-Pereira, Dirceu Solé

**Affiliations:** a Pontificia Universidade Católica do Rio Grande do Sul (PUC-RS), Porto Alegre, RS, Brasil; b Universidade Federal de São Paulo (UNIFESP), São Paulo, SP, Brasil

**Keywords:** Vitamina D, Gestação, Lactação, Feto, Recém-nascido, Criança, Programação fetal

## Abstract

**OBJETIVO:**

Deficiência de vitamina D (DVD) nas gestantes e seus filhos é problema de saúde, com consequências graves à saúde de ambos. Assim, esta revisão visou reavaliar a magnitude e as consequências da DVD na gestação, lactação e infância, fatores de risco associados, métodos de prevenção, além de explorar os mecanismos epigenéticos na vida fetal capazes de explicar benefícios não-esqueléticos da vitamina D (ViD).

**FONTES DE DADOS:**

Selecionaram-se artigos originais, de revisão e consensos com nível elevado de evidência para decisões clínicas relacionadas à DVD na saúde das gestantes e seus filhos e artigos sobre sua ação sobre os mecanismos epigenéticos da programação fetal de doenças crônicas na vida adulta, publicados no PubMed nos últimos 20 anos, empregando-se *VitD status*, e em combinação com *Pregnancy, Offspring health, Child outcomes* e *Programming*.

**SÍNTESE DOS DADOS:**

Abordou-se fisiologia, metabolismo, fatores de risco para a DVD e implicações na gravidez, lactação e infância, concentração de corte para definir DVD, variabilidade de métodos na sua detecção, recomendações sobre a reposição de ViD nas gestantes, no recém-nascido e na criança, bem como sobre ter as influências epigenéticas da ViD.

**CONCLUSÕES:**

DVD é frequente entre gestantes de alto risco e seus filhos. Monitorar rotineiramente os níveis séricos de 25(OH)D_3_ no período antenatal é imperativo. Medidas preventivas precoces devem ser instituídas à menor suspeita de DVD na gestante, para reduzir morbidades durante a gestação e a lactação, bem como seu posterior impacto sobre o feto, o recém-nascido e na infância.

## Introdução

A deficiência de vitamina D (DVD) é apontada como um problema de saúde pública em muitos países, e as gestantes têm sido identificadas como um grupo de alto risco, em quem a prevalência de DVD oscila entre 20-40%.^1^

Apesar de reconhecer-se que a suplementação com vitamina D (ViD) é efetiva na prevenção da DVD, muitas crianças nascem com deficiência o que gera questionamentos: como e porque a DVD afeta a gestação, o feto e a saúde o recém-nascido?^2^

O aumento no número de estudos sobre este tema revela resultados conflitantes sobre a associação entre os níveis de 25(OH)D na gravidez e os efeitos adversos para a saúde materna e fetal, esqueléticos e não esqueléticos (doenças autoimunes, doenças cardiovasculares, diabete e certos tipos de câncer via "fetal imprinting").^3^ Assim, é oportuno rever a DVD nas mães e seus filhos para que sejam implantadas estratégias na sua prevenção na gestação, na lactação, com o intuito de evitar seu impacto sobre o feto, o recém-nascido e na infância, visando uma possível redução no desenvolvimento futuro de doenças crônicas na idade adulta.

## Método

Para a seleção dos artigos empregados na presente revisão, foi utilizada a base de dados do PubMed, avaliando-se como período de busca os últimos 20 anos. Foram empregados os seguintes termos de busca: *VitD status* isolado e em combinação com os termos: *Pregnancy, Offspring health, Child outcomes*, *Programming.* Entre os trabalhos encontrados foram excluídos os de relato de caso, os de intervenção sem randomização, sendo selecionados artigos originais, artigos de revisão e consensos com nível elevado de evidência para decisões clínicas relacionadas à DVD na saúde das gestantes e seus filhos. Selecionaram-se também os artigos que avaliaram a influência da ViD nos mecanismos epigenéticos da programação fetal de doenças crônicas na vida adulta, focalizando-se nos mais recentes. Os artigos mais relevantes segundo os objetivos da presente revisão foram os escolhidos.

## Fisiologia e metabolismo da vitamina D

Para o ser humano, existem duas fontes de ViD. Uma exógena fornecida pela dieta sob a forma de vitamina D_2_ e D_3_. Na produção endógena, o colecalciferol (D_3_), principal fonte de ViD, é sintetizado na pele por ação da radiação ultravioleta B (UVB) pela fotolise do 7-dehidrocolesterol e transformação em vitamina D_3_. A exposição suficiente à luz solar ou radiação UVB é a de até 18UI/cm^2^ em 3 horas. Este processo transcorre em duas etapas: a primeira acontece nos estratos profundos da derme e consiste na foto conversão do 7-dehidrocolesterol em pré-vitamina D ou pré-calciferol (fig. 1).^4^

**Figura 1:** Síntese e metabolismo da vitamina D assim como sua ação na regulação dos níveis de cálcio, fósforo e metabolismo ósseo.8 UVB, raios ultravioleta B; 25(OH)D, 25-hidroxivitamina D; 1,25(OH)2D, 1,25-di-hidroxivitamina D; FGF-23, fator de crescimento de fibroblastos 23; Ca2+, ions cálcio; HPO42-, íons fósforo.

Na segunda etapa, há uma isomerização química dependendo da temperatura corporal, e a pré-vitamina D se transforma lenta e progressivamente em vitamina D_3_ que tem alta afinidade pela proteína transportadora de ViD (DBP) e a pré-vitamina D, com menor afinidade de ligação, permanece na pele.^4^ Ao chegar à rede capilar cutânea, a ViD é transportada ao fígado, unida à DBP, onde inicia sua transformação metabólica.^4^

Os dois tipos de ViD sofrem processamentos complexos para serem metabolicamente ativos.^5^ Inicialmente o pré-hormônio sofre hidroxilação no fígado, no carbono 25 por ação da 25-vitamina D 1a-hidroxilase (1-OHase), que compõe um sistema enzimático dependente do citocromo P-450 (CYP27B) presente nas mitocôndrias e microssomas hepáticos e origina a 25-hidroxi-vitamina D (25(OH)D), a forma circulante mais abundante de ViD.^4^ Sua concentração sanguínea média é 20-50 ng/ml (50-125 nmol/L) e tem vida média de aproximadamente 3-4 semanas.^4^ Estima-se que seu *pool* circulante esteja em equilíbrio dinâmico com as reservas de 25(OH)D (músculo e tecido adiposo), o que torna a concentração sanguínea fidedigna do estado das reservas de ViD no organismo. 4 Em situações de normalidade, a percentagem de conversão a 25(OH)D é baixa, com distribuição de quase 50% nos compartimentos gorduroso e muscular. Havendo ingestão em excesso de ViD, a maior parte é fixada nos depósitos graxos. 4

Como a 25(OH)D possui baixa atividade biológica, é transportada ao rim onde sofre a segunda hidroxilação, obtendo-se as formas ativas: o calcitriol (1a-dihidroxivitamina D) (1,25(OH)_2_D) e a 24,25 dihidroxivitamina D (24,25(OH)_2_D) pela ação respectiva das enzimas 1-OHase e da 24-vitamina D-hidroxilase (24-OHase), presentes nas mitocôndrias das células do túbulo contornado proximal.^5^

A DBP e a 25(OH)D são filtradas pelo glomérulo e absorvidas no túbulo proximal por receptores de lipoproteína de baixa densidade, que regulam a captação do complexo 25(OH)D-DBP dentro das células do tubo e a posterior hidroxilação a 1,25(OH)_2_D.^4^

A 1-OHase é encontrada também em outros tecidos que expressam receptores de ViD, como placenta, cólon, células mononucleares ativadas e osteoblastos que poderiam produzir 1,25(OH)_2_D com função local autócrina ou parácrina.^6^

Numerosos fatores regulam os níveis de 1,25(OH)_2_D: a 1-OHase cuja hidroxilação é ativada pelo paratohormônio (PTH) e a calcitonina, e é inibida pelos níveis plasmáticos de cálcio, fósforo e da própria 1,25(OH)_2_D cuja vida média é de 15 dias.^6^

A concentração sanguínea de fósforo atua de forma direta, sem intervenção do PTH, a hipofosfatemia aumenta a produção de 1,25(OH)_2_ D.

Assim, além da principal ação da ViD em manter os níveis fisiológicos de cálcio e fósforo capazes de permitir o metabolismo, a transmissão neuromuscular e a mineralização óssea, tem sido descrita a presença de receptores de ViD no osso, medula óssea, cartilagem, folículo piloso, tecido adiposo, suprarrenal, cérebro, estômago, intestino delgado, túbulo renal distal, colon, pâncreas (células B), fígado, pulmão, músculo, linfócitos B e T ativados, coração, células do músculo liso vascular, gônadas, próstata, retina timo e tireoides, o que reforçam funções tão diversas e importantes para a ViD (fig. 2).^5^ A figura 3 resume os mecanismos envolvidos no controle dos níveis séricos de cálcio e fósforo.^7^

**Figura 2:** Funções não esqueléticas da 1,25-dihidroxivitamina D. Adaptado de Holick MF et al.8 VDR, receptor da vitamina D; RXR, região alvo; 1-OHase, 25-hidroxivitamina D-1a-hidroxilase; 25(OH)D, 25-hidroxivitamina D; 1,25(OH)2D, 1,25-di-hidroxivitamina D; 24-OHase, 25-hidroxivitamina D-24-hidroxilase; p21 e p27, genes envolvidos no controle da proliferação, inibição da angiogênese e apoptose celulares.

**Figura 3:** Mecanismos regulatórios dos níveis séricos de cálcio e fósforo. Adaptado de Ross AC et al.7 PTH, hormônio da paratireoide; Ca2+, íon cálcio; PO43-, íons fosfato; ↑, aumento; ↓, redução.

## Fatores de risco para deficiência de vitamina D

A principal fonte de ViD para crianças e adultos é a exposição à luz natural, portanto a principal causa da DVD é a diminuição da sua produção endógena. Qualquer fator que afete a transmissão da radiação UVB ou interfira com a sua penetração na pele determinará a diminuição de 25(OH)D.^4^

- Uso de protetor solar com fator de proteção 30 reduz a síntese de ViD na pele, acima de 95%.
- Pessoas com pele mais escura têm uma proteção natural ao sol, pois a melanina absorve a radiação UVB e necessitam de um tempo de exposição solar 3 a 5 vezes maior para sintetizar a mesma quantidade de ViD que as de pele clara.
- O envelhecimento da pele e a idade diminuem a capacidade cutânea da produção da ViD, por menor disponibilidade de 7-dehidrocolesterol.
- Os danos na pele como queimadura diminuem a produção de ViD.
- A contaminação atmosférica e a nebulosidade podem atuar como filtro solar.
- A estação do ano e, a hora do dia influi dramaticamente na produção cutânea de ViD.

A segunda causa é a ingestão reduzida de ViD, pois poucos alimentos contém quantidades elevadas de ViD (peixes azuis, gema de ovo). O aumento da ingestão de ViD pode ser com produtos fortificados como os derivados lácteos, embora a quantidade de ViD neles presente pode ser insuficiente para um estado adequado de ViD.^8^

A obesidade também pode associar-se à DVD, pois a ViD, lipossolúvel, é sequestrada pela gordura corporal. Outro fator é a má absorção de gorduras, como ocorre como o uso de quelantes dos ácidos biliares (colestiramina), na fibrose cística, na doença celíaca, na doença de Crohn, entre outras etc...^8^ Além disso, medicamentos anticonvulsivantes, glicocorticoides, e para tratamento para HIV por aumentarem a expressão hepática do citocromo P-450 e o catabolismo de 25(OH)D. Na insuficiência hepática grave, doença crônica granulomatosa, alguns linfomas e no hipoparatireoidismo primário, os pacientes têm o metabolismo de 25(OH)D a 1,25(OH)_2_D aumentado e assim alto risco de DVD.^8^

## Deficiência de vitamina D na gravidez e programação fetal

Durante a vida fetal os tecidos e órgãos do corpo passam por períodos críticos de desenvolvimento que coincidem com fases de rápida divisão celular.^9^ A programação fetal é um processo pelo qual um estímulo ou insulto, durante um determinado período de desenvolvimento teria efeitos ao longo da vida,^10^ esse termo é usado para descrever os mecanismos que determinam a adaptação fetal a essas mudanças que acompanham a interação gene-ambiente durante períodos específicos do desenvolvimento fetal.^9^

Demonstra-se que exposições ambientais e nutricionais durante esses períodos sensíveis da vida podem influenciar o crescimento fetal e o desenvolvimento das funções fisiológicas de órgãos e sistemas. Alterações permanentes em muitos processos fisiológicos dessa programação podem modificar os padrões de expressão de genes com influências sobre fenótipos e funções (mecanismos epigenéticos).^11^

Assim, quanto mais próximas da fertilização essas modificações acontecerem, maior o potencial para que alterações epigenéticas e sua correspondência no recém-nascido ocorram em resposta a alterações ambientais. Essas mudanças na placenta/embrião/feto fornecem uma explicação plausível para o conceito da origem fetal das doenças no adulto.^12^

Reconhece-se hoje que a nutrição no início da vida e outros fatores ambientais desempenham um papel-chave na patogênese e na predisposição de doenças, que parecem se propagar a gerações subsequentes. As modificações epigenéticas estabelecem uma ligação com o estado nutricional durante períodos críticos do desenvolvimento e provocam mudanças na expressão gênica que podem levar ao desenvolvimento de fenótipos de doenças.^13^

Evidências recentes apontam que os nutrientes podem modificar a programação imunológica e metabólica durante períodos sensíveis do desenvolvimento fetal e pós-natal. Assim, padrões modernos de dieta poderiam aumentar o risco de desregulação imunológica e metabólica associado ao incremento de uma ampla gama de doenças não comunicáveis.^11^ Entre esses nutrientes destaca-se a ViD, cujos efeitos sobre a programação fetal e a regulação dos genes poderiam explicar porque ela tem sido relacionada a tantos benefícios para a saúde ao longo da vida.^8^^,^ 14 ^and^ 15

Parece haver uma janela de desenvolvimento precoce na vida que pode moldar a natureza da resposta imunológica na vida adulta, assim eventos precoces na vida que predispõem os indivíduos a doenças pulmonares crônicas, não estariam limitados somente ao período pós-natal, evidências indicam que existem efeitos intrauterinos como o tabagismo materno, a dieta, a ViD, que influenciam no desenvolvimento do pulmão e o posterior desenvolvimento de asma e doença pulmonar obstrutiva crônica.^16^ ^and^ 17

Como grande parte da reprogramação que ocorre durante a infância pode passar despercebida até a idade adulta, o melhor entendimento da interação entre a genética e a epigenética em janelas de tempo crítico do desenvolvimento, melhorariam nossa capacidade de determinar a suscetibilidade individual para uma ampla gama de doenças.^13^ Embora essas mudanças epigenéticas pareçam ser potencialmente reversíveis, pouco se sabe ainda sobre a velocidade e a extensão das melhorias em resposta às modificações positivas do ambiente, incluindo a nutrição, assim como ainda se desconhece o quanto elas são dependentes da duração da exposição a um meio ambiente materno deficiente.^18^

Assim, pode-se observar que, apesar de todo esse novo leque de informações, a nutrição materna tem recebido pouca atenção no contexto de implantação de metas efetivas de prevenção (*MDG, Millennium Development Goals*). Isto poderia ser atribuído à falta de uma base sólida e forte para justificar o enorme esforço necessário para aprimorar o estado nutricional de todas as mulheres em idade reprodutiva. 19 Elucidar o verdadeiro papel da epigenética nutricional 13 ^and^ 14 na programação fetal das mulheres grávidas, especialmente aquelas com DVD, permitiria a utilização de medidas de prevenção eficazes destinadas a melhorar a saúde materna e fetal e evitar o desenvolvimento de futuras doenças crônicas.

## Metabolismo da vitamina D e cálcio na gravidez

Durante a gravidez e a lactação, ocorrem mudanças significativas no metabolismo do cálcio e ViD para prover as necessidades daquele na mineralização óssea fetal. No primeiro trimestre, o feto acumula 2-3 mg/dia de cálcio no esqueleto, que dobra no último trimestre.^1^

A gestante se adapta às necessidades fetais e aumenta a absorção de cálcio no início da gravidez, atingindo um nível máximo no último trimestre.^1^ A transferência é contrabalançada pelo aumento da absorção intestinal e redução da excreção urinária de cálcio.

Os níveis plasmáticos de 1,25(OH)_2_D aumentam no início da gravidez, chegando ao máximo no terceiro trimestre, e voltando ao normal durante a lactação. O estímulo para o aumento da síntese de 1,25(OH)_2_D não é claro, porque os níveis de PHT não mudam durante a gestação.^1^

Um estímulo potente à transferência placentária de cálcio e síntese placentária de ViD é o peptídeo relacionado ao PTH (PHTrP), produzido na paratireoide fetal e nos tecidos placentários e que aumenta a síntese de ViD.^1^ O PTHrP pode atingir a circulação materna e atua através do receptor de PTH/PTHrP no rim e no osso, sendo mediador do aumento da 1,25(OH)2D e auxiliando na regulação dos níveis de cálcio e do PTH na gestante.^1^

Outros sinais envolvidos nessa regulação incluem a prolactina e o hormônio lactogeno placentário, que aumentam a absorção intestinal de cálcio, diminuem a excreção urinária de cálcio e estimulam a produção de PHTrP e 1,25(OH)_2_D. Também o aumento dos níveis sanguíneos maternos de calcitonina e osteoprotegina protegem o esqueleto materno da reabsorção excessiva de cálcio.^1^

Além disso, durante a lactação, há deficiência relativa de estrógeno decorrente da elevação dos níveis de prolactina, o que determina a reabsorção óssea e a supressão dos níveis de PHT. Os níveis de PHTrP estão elevados e atuam como um substituto do PHT, continuando a absorção de cálcio urinário e a reabsorção óssea.^1^

## Implicações da deficiência de vitamina D na gestação

Estudos recentes enfatizam a importância de papeis não clássicos da ViD na gestação e na placenta e relacionam a DVD na gravidez à: pré-eclampsia, resistência à insulina, diabete gestacional, vaginose bacteriana e aumento da frequência de parto cesáreo.^20^

A suplementação com ViD reduz o risco de pré-eclampsia. Estudos em mulheres com pré-eclâmpsia têm demonstrado baixa excreção urinária de cálcio, níveis baixos cálcio ionizado, níveis altos de PTH e níveis baixos de 1,25(OH)_2_D.^21^ Documentou-se, também, relação entre DVD materna (<50 nmol/L) e aumento do risco de diabete gestacional (OR:2,66; IC95%:1,01-7,02),^22^ como também ser a DVD fator de risco independente de vaginose bacteriana na gravidez.^23^ Estudo randomizado e controlado recente demonstrou que a suplementação com 4.000 UI/d durante a gravidez, associou-se à redução do risco de morbidades combinadas como: infecções maternas, parto cesáreo e parto prematuro.^21^^,^ 22 ^,^ 23 ^and^ 24

Estudo prospectivo demonstrou ser o parto cesáreo quatro vezes mais comum em mulheres com DVD (<37,5 nmol/L) em comparação a mulheres com valores normais (OR:3,84; IC95%:1,71-8,62).^25^

## Implicações da deficiência de vitamina D na lactação e infância

Níveis adequados de ViD são também importantes para a saúde do feto e do RN e a mineralização deficiente do esqueleto *in utero* decorrente da DVD pode manifestar-se no RN por: raquitismo congênito, crânio tabes ou osteopenia. 1

A DVD materna é um dos principais fatores de risco de DVD na infância, pois, nas primeiras 6-8 semanas de vida os RN dependem da ViD transferida pela placenta no útero materno. Essa relação é linear^26^ e os níveis de 25(OH)D do RN correspondem a 60-89% dos valores maternos.^2^

Esses níveis diminuem na oitava semana, e, portanto, os lactentes amamentados exclusivamente têm risco elevado de DVD, pois o leite humano tem baixa concentração de ViD (aproximadamente 20-60 IU/L; 1,5-3% do nível materno). Essa concentração não é suficiente para manter níveis ótimos de ViD, sobretudo quando a exposição solar é limitada,^27^ podendo induzir convulsões por hipocalcemia e miocardiopatia dilatada.^1^

Estudos observacionais demonstraram que os níveis baixos de ViD na gestação e a DVD na infância estão relacionados ao aumento de outras manifestações não esqueléticas,^2^ como a maior incidência de infecções agudas do trato respiratório inferior e sibilâncias recorrentes nos primeiros cinco anos de vida.^28^

Resultados contraditórios são observados com relação ao aumento do risco de das enfermidades alérgicas asma, eczema e rinite na DVD.^29^ Entretanto, estudo de coorte documentou aumento de asma e eczema entre as crianças cujas mães tinham níveis séricos altos de 25(OH)D durante a gravidez.^30^

Escolares japoneses suplementados com ViD (1200 UI/d) tiveram redução de 42% na incidência de Influenza tipo A.^1^ Estudo de coorte demonstrou que a suplementação com 2000 UI/d de ViD, durante o primeiro ano de vida, associou-se a redução da incidência de diabete tipo I durante o seguimento de 30 anos.^31^

Esses resultados demostram a importância da manutenção de níveis adequados de ViD na vida fetal e nos primeiros anos de vida e na infância.

## Deficiência, insuficiência e suficiência de vitamina D

Questiona-se o ponto de corte para definir o estado de ViD em base aos valores de 25(OH)D. Atualmente existem dois critérios:

O Comitê do Instituto de Medicina (IOM, Estados Unidos)^32^ considera valores inferiores a 20ng/ml (50nmol/L) como indicadores de DVD sendo 10ng/ml (25nmol/L) DVD grave e de 10-19ng/ml (25-49nmol/L) insuficiência. Concentrações inferiores a 10 ng/ml associam-se a raquitismo e osteomalácia, em crianças e adultos. Já entre 10-19ng/ml há aumento da reabsorção óssea e risco elevado de hipoparatireoidismo secundário. Por isso a IOM recomenda uma concentração limite de 20 ng/ml como adequada para manter a saúde óssea em todas as idades.

A *Endocrine Society* (Estados Unidos) propõe a DVD com concentrações inferiores a 20ng/ml e a insuficiência às entre 20-30ng/ml (50-75nnol/L). 33 Na prática clínica um paciente teria nível suficiente, quando a concentração de 25(OH)D fosse superior a 30ng/ml. Numerosos autores apoiam esta concentração de corte para a saúde musculoesquelética e do metabolismo mineral (prevenção do raquitismo e da osteomalácia, níveis elevados de PTH, fraturas por osteoporose e quedas em pessoas mais velhas). 34

As principais diferenças entre a IOM^32^ e a *Endocrine Society* 33 estão nos pontos finais da saúde em geral. A IOM faz recomendações para garantir a saúde do esqueleto e sugere haver falta de evidências para que recomendações dos possíveis benefícios não esqueléticos da ViD sejam feitas, considerando que os indivíduos com valores inferiores a 20ng/ml não são deficientes, já que 97% dos sujeitos com esses níveis têm uma saúde óssea adequada. 32

A *Endrocrine Society* 33 considera que os níveis séricos superiores a 30ng/ml proporcionam maiores benefícios para a saúde em geral, comparado aos de 20ng/ml e que a saúde do esqueleto não está garantida com os valores inferiores a 30ng/ml, dados apoiados por três supostas observações:

A elevação de PTH tem uma meseta quando o 25(OH)D sérico é igual ou maior que 30ng/ml.

Há redução do risco de fraturas nas pessoas maiores com níveis iguais ou superiores a 30ng/ml.

A absorção do cálcio é máxima para os níveis séricos de 30ng/ml.

## Método de detecção

As concentrações séricas de 25(OH)D são o melhor indicador do estado de ViD. Entretanto, questões metodológicas limitam a comparação entre os estudos assim como a adoção de pontos de corte para definir a hipovitaminose D.^4^

Diante do método empregado é importante considerar-se: 1) O método quantifica o nível real de ViD? e se 2) Estes resultados são reprodutíveis e comparáveis entre os laboratórios?^4^

A Cromatografia Líquida Acoplada à Espectometria de Massas (LC-MS/MS) foi recomendada como o método preferido pelo *National Diet and Nutricion Survey.* 34 Em linhas gerais, todos os métodos disponíveis são válidos para detectar DVD graves. Já para as moderadas, há risco de erro que pode ser diminuído considerando-se os valores de referência de cada laboratório, entretanto, para estudos de investigação, os métodos empregados devem ser padronizados. 6

## Recomendações

Se a principal fonte de ViD advém da exposição solar, é difícil estabelecer-se requerimentos generalizados da sua ingestão, especialmente pelas muitas variáveis associadas à sua deficiência.^6^

Na tabela 1 são apresentadas as diferentes doses diárias recomendadas de ViD**.** Embora o IOM recomende a ingestão diária de 200UI de ViD, esta mostrou-se insuficiente para manter concentrações de 25(OH)D acima de 50nmol/L.^35^ Por outro lado, mesmo reconhecendo que a exclusão de exposição solar habitual é um risco para a DVD, desconhece-se qual o nível de exposição segura e suficiente para manter níveis adequados de ViD.^35^ Na tabela 2 são apresentados os diferentes conteúdos de alimentos fortificados ou não com ViD disponíveis para consumo nos Estados Unidos da América do Norte. No Brasil, tais dados são escassos e na maioria das vezes não refletem o conteúdo de todos os alimentos industrializados disponíveis.

**Tabela 1:** Ingestas dietéticas de referência e ingestas máximas toleráveis de Vitamina D em diferentes etapas da vida - IOM, 201032

Idade	Vitamina D mcg/dia			
	IA	REM	IDR	NMIT
Lactentes (0 a 6 meses)	10 (400 UI)	-	-	25 (1000 UI)
6 a 12 meses	10 (400 UI)		15 (600 UI)	38 (1520 UI)
13 meses a 3 anos	-	10 (400 UI)	15 (600 UI)	63 (2520 UI)
4 a 8 anos	-	10 (400 UI)	15 (600 UI)	75 (3000 UI)
Homens: 9 a 70 anos	-	10 (400 UI)	20 (800 UI)	100 (4000 UI)
Mulheres: 9 a 70 anos	-	10 (400 UI)	15 (600 UI)	100 (4000 UI)
Maiores de 70 anos	-	10 (400 UI)	20 (800 UI)	100 (4000 UI)
Gravidez (14 a 50 anos)	-	10 (400 UI)	15 (600 UI)	100 (4000 UI)
Lactação (14 a 50 anos)	-	10 (400 UI)	15 (600 UI)	100 (4000 UI)

IA, ingesta adequada; REM, requerimento estimado médio; IDR, Ingesta diária recomendada; NMIT, Nível máximo de ingesta tolerável; entre parênteses o valor correspondente em unidades internacionais (UI).

**Tabela 2:** Fontes alimentares de Vitamina D2 e Vitamina D37

Fonte	Conteúdo de Vitamina D
*Salmão*	
Selvagem – 100 g	600-1000UI de Vit D_3_
Criado em cativeiro – 100 g	100-250 UI Vit D_3_ ou D_2_
Enlatado – 100g	300-600 UI Vit D_3_
*Sardinha enlatada – 100g*	~ 300 UI Vit D_3_
*Cavalinha enlatada – 100g*	~ 250 UI Vit D_3_
*Atum enlatado – 100g*	~ 230 UI Vit D_3_
*Óleo de fígado de bacalhau (1 colher sopa)*	~ 400-1000 UI Vit D_3_
*Cogumelo Shiitake fresco – 100g*	~ 100 UI Vit D_3_
*Cogumelo Shiitake seco – 100g*	~ 1600 UI Vit D_3_
*Gema de ovo*	~ 20 UI Vit D_3_ ou D_2_
*Alimentos fortificados*	
Leite fortificado - 240 mL	~ 100 UI Vit D_3_
Suco de laranja - 240 mL	~ 100 UI Vit D_3_
Fórmulas infantis - 240 mL	~ 100 UI Vit D_3_
Iogurtes fortificados - 240 mL	~ 100 UI Vit D_3_
Manteiga fortificada – 100g	~ 50 UI Vit D_3_
Margarina fortificada – 100g	~ 430 UI Vit D_3_
Queijos fortificados – 85g	~ 100 UI Vit D_3_
Cereais fortificados - porção refeição	~ 100 UI Vit D_3_

Na maioria dos países, o monitoramento dos níveis séricos de 25(OH)D durante a gravidez não é realizado, entretanto, é recomendado que mulheres com um ou mais fatores de risco para DVD sejam monitorizadas no início e na metade da gestação.^36^ Assim, reduzir-se-ia o risco da DVD na gestação e os efeitos negativos sobre a mãe e o feto, entretanto, a dose apropriada de suplementação de ViD para a gestante com a finalidade de evitar DVD é desconhecida.

Há poucos estudos que avaliaram a suplementação de ViD na gravidez, assim como os níveis ótimos a serem oferecidos. Vários fatores dificultam a observação de dose-resposta adequada entre níveis baixos de 25(OH)D e os resultados clínicos: falta de dados com níveis séricos extremos de 25(OH)D e grande variedade de indivíduos estudados (diversidade de locação, latitude, estação, etnia, índice de massa corpórea, tipo de dieta, estilo de vida, pigmentação da pele, história familiar de complicações metabólicas na gravidez, atividade física e método usado para quantificar 25(OH)D).^1^

Uma metanálise de estudos em adultos em suplementação de ViD (2000UI/d) e saúde óssea demonstrou que, para cada 1UI de vitamina D3 ingerida, corresponde aumento de 0,016nmol/L nos níveis séricos de 25(OH)D.^37^ Apesar de evidências limitadas sobre os efeitos da suplementação de ViD na gravidez e os resultados na saúde da mãe, nos efeitos perinatais e na infância, a suplementação de ViD (800-1000UI/d) acompanhou-se de efeito protetor aos RN com baixo peso.^9^ ^and^ 38

A *Canadian Academy of* Pediatrics_37_ recomenda suplementar com 2000UI/d durante a gravidez e lactação. 38 Segundo o *American College of Obstetricians and Gynecologists*_38_ na presença de DVD diagnosticada durante a gestação deverá ocorrer a reposição com 1000-2000UI/dia de ViD.

Estudos demonstram que a exposição materna na gravidez a concentrações séricas de 25(OH)D acima de 75nmol/L não influenciam na inteligência e saúde psicológica das crianças ou no sistema cardiovascular, mas poderia aumentar o risco de doenças atópicas.^30^

Em resumo, os níveis séricos de ViD na gravidez são uma grande preocupação e a prevenção da DVD, na gestante e nos seus RN é vital e urgente.

## Recomendações de vitamina D para recém-nascido e crianças

A *Canadian Academy of Pediatrics* define as necessidades de ViD durante o primeiro ano de vida como: 200 UI/d para RN prematuros e 400 UI/d para as outras crianças. Todavia, o incremento de peso observado no primeiro ano de vida seria acompanhado por aumento nas necessidades de ViD de modo peso dependente? 38 Além disso, recomenda também que lactantes e crianças sejam expostas ao sol por períodos curtos, provavelmente menores de 15 minutos. 38

A *American Academy of Pediatrics* recomenda que toda criança amamentada exclusivamente ao seio, deva receber suplementação com 400UI/dia de ViD logo após o nascimento e continuar a ingestão durante seu desenvolvimento até a adolescência. 5 Preocupados com a saúde óssea de RN prematuros, recomendam o monitoramento bioquímico dos seus níveis de 25(OH)D durante a internação, e recomendam 200-400 UI/d de ViD, tanto durante a internação como após a alta hospitalar. 39 ^and^ 40 Recentemente, IOM recomendou 400 UI/d para crianças menores de 1 ano e 600UI/d para as entre 1-8 anos. 32

## Conclusão

A DVD nas gestantes e seus filhos é importante problema de saúde, com potenciais consequências adversas à saúde global. Estratégias de prevenção devem assegurar a suficiência de ViD nas mulheres durante a gravidez e a lactação. Intervenções baseadas em evidências para melhorar a nutrição materna e fetal, como de ViD, se acompanham de redução do seu impacto na saúde de seus filhos.^41^

As ambiguidades entre a definição do estado de ViD combinado à falta de consistência nas recomendações relacionadas a: incorporação do teste de rotina de 25(OH)D no pré-natal, principalmente nas gestantes com fatores de risco para DVD, dose e idade gestacional para o início da suplementação de ViD, pontos de corte universais para valores normais de ViD, a falta de educação sobre os benefícios da ViD e da necessidade de uma exposição solar adequada, representam barreiras importantes no avanço da implantação de guias de suplementação de ViD, para melhorar EM curto prazo este importante problema de saúde nas gestantes e seus filhos. São necessários estudos de larga escala, em diversas localidades geográficas para identificar o verdadeiro papel da ViD na saúde das gestantes e no "fetal imprinting" dos seus filhos.
